# The stability of ethnic identity in England and Wales 2001–2011

**DOI:** 10.1111/rssa.12175

**Published:** 2016-01-27

**Authors:** Ludi Simpson, Stephen Jivraj, James Warren

**Affiliations:** ^1^ University of ManchesterUK; ^2^ University College LondonUK; ^3^ Advisory, Conciliation and Arbitration ServiceLondonUK

**Keywords:** Census, England and Wales, Ethnicity, Identity, Office for National Statistics Longitudinal Study, Reliability

## Abstract

The instability of ethnicity measured in the national census is found to have doubled from the period 1991–2001 to the period 2001–2011, using the Longitudinal Study that links a sample of individuals’ census records across time. From internal evidence and comparison with results from the Census Quality Survey and the Labour Force Survey, estimates are made of instability due to changing question wording, imputation of missing answers, proxy reporting, recording errors and changes in the allocation of write‐in answers. Of the remaining instability, durable changes of ethnicity by individuals are thought to be considerably less common than changes due to a person's sense of identity not closely fitting the categories offered in the census question. The instability creates a net change in size of some ethnic groups that is usually small compared with the change in population between censuses from births, deaths and migration. Consequences for analysis of census aggregate and microdata are explored.

## 1 Aims and structure

Although it is widely accepted that the definition and measurement of ‘ethnicity’ is shaped culturally, its use in official statistics in several developed countries encourages a practical use of the statistical products about ethnicity as objective measures of social groups with clear boundaries. In this paper we examine the reliability of measuring ethnicity in a national census, using the Longitudinal Study (LS) of England and Wales which, for a 1% sample of the population, links individuals’ answers to successive decennial censuses. We look for change over time in recorded ethnic group, and therefore we focus on its reliability rather than validity.

We believe that our analyses will be of interest in many contexts beyond the census, and in many countries beyond England and Wales, because they probe the meaning and measurement of ethnicity. However, our prime motivations are to support analysts of ethnic group data by answering five questions, which occupy Sections 4, 5, 6, 7, 8. How much turnover of each ethnic group's population occurs between two censuses? For those who are enumerated in both censuses, to what extent is each group comparable from one census to the next? What are the sources of unreliability of ethnic group responses: how much is due to change in the question, to errors of transcribing, to shifts in identity, to ambiguity in the question such that a specific ethnic identity may fit more than one category and to the imputation of missing data? How successful is that imputation? And, finally, what are the variables associated with unreliability of recorded ethnic group? Our answers lead to practical recommendations about the groups that can best be compared over time, the lack of consistent shifts in ethnicity when projecting future population composition and the treatment of missing data in microanalyses.

**Figure 1:**
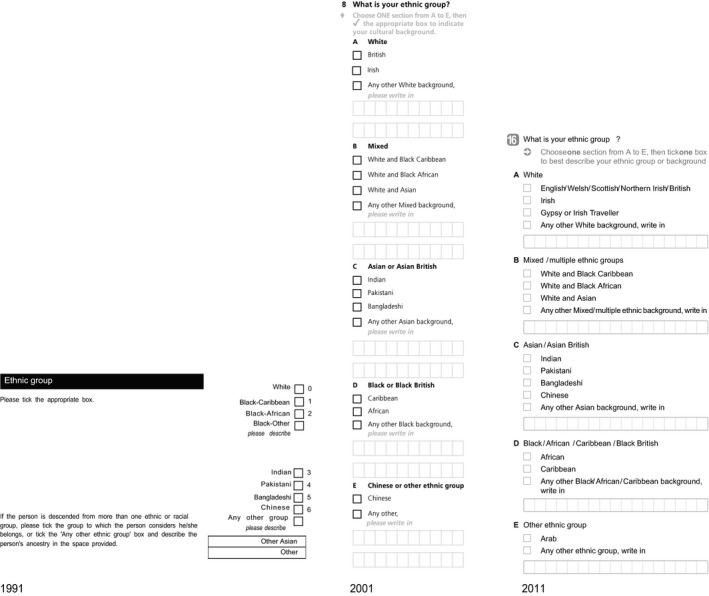
Ethnic group question used in the population censuses of England and Wales: for 2011 in England, the first category under ‘White’ was labelled ‘English/Welsh/Scottish/Northern Irish/British’; it is usually abbreviated to ‘White British’ in the text

Our answers to these questions rely on the Office for National Statistics (ONS) LS linked responses from the 1991, 2001 and 2011 censuses for a sample in England and Wales. These are the only three censuses in the UK that have included a question on ethnicity (Fig. 1). During this time, the population has become more ethnically diverse (Jivraj and Simpson, 2015), such that 10.9 million or 19.5% of England and Wales residents were recorded as one of the 17 ethnic minority groups in 2011. We focus on new results from the LS's link between the 2001 and 2011 censuses.

Sections 2 and 3 review previous literature and describe the data. We finish in Section 8 with a discussion of our results and their implications not only for analysts of official data but also for the development of ethnic group questions in the UK and elsewhere.

## 2 Previous analyses of the validity and reliability of measured ethnicity

The second era of global migration (Livi‐Bacci, 2012) since the Second World War has expanded the measurement of indigenous and migrant populations, using terminology of descent, cultural origins, heritage and race (e.g. Christopher (2005), Morning (2008) and Mateos (2014)). The European Commission recognizes that the scarcity of ethnic data in most Member States might hinder proper monitoring of the application of European Community legislation on combating discrimination. It implies a desire to reduce its member countries’ resistance to official measurement of ethnicity or religion, which is a result of secular republicanism and horror of the official targeting of religion in the 20th century fascist epoch in Europe (Simon, 2012).

In countries where ethnicity has been officially collected, categories of ethnicity have developed according to changing policy priorities, and as streams of migration have diversified. In Britain, the desire for information to implement a Race Relations Act led to the first census ethnic group question in 1991, and its development in the 2001 and 2011 censuses (Bulmer (1996), Finney and Simpson (2009), pages 23–45, and Aspinall (2007)). In England and Wales, the original 10 output categories have been expanded in number to 18. The question is now structured as several country or regional categories within each of three headings denoting skin colour or appearance (‘White’, Asian and ‘Black’), a ‘Mixed’ heading, and an ‘Other’ heading. Under each of these five headings, an ‘Other’ category allows responses to be written in that do not fit the specified categories. These responses may be reallocated by the census office (Nigerian written under ‘Black Other’ would be reallocated to African) or left as a residual (American would be left as Black Other). Scotland and Northern Ireland have developed slightly different approaches (Jivraj and Simpson (2015) reproduced each question in the UK since 1991). In 2011 in England and Wales, there were related questions on birthplace (but not parental birthplace), religion, language, passports held, national identity and the year of most recent immigration for those who were not born in the UK.

Other countries have developed questions related to ethnicity in different ways according to their cultural histories and political priorities, as reviewed in Morning (2008), Simon (2012) and Christopher (2005).

To assess the validity of questions about ethnicity many studies have focused on the questions’ acceptability to respondents (Pearce and Thomas, 1990; Office for National Statistics, 2009; Compton *et al*., 2013). No question that is unacceptable to a significant section of respondents will be practical to implement. A comprehensive study of the validity of questions about ethnicity would also attempt to match the question to its intended purposes to support government policies related to race relations, discrimination, immigration and service delivery. We focus on a third specific aspect of validity, namely the reliability of the responses: does an individual provide the same response when the ethnicity question is asked on more than one occasion?

Discordance between ethnicity recorded on the census and on administrative records has been measured for England. Responses in the 2011 census were matched to 6.7 million schoolchildren's school records from January 2011, and their ethnic group responses compared (Office for National Statistics, 2014a). State schools are allowed to record ethnicity with more detail than used in the census but could be aggregated to 17 categories that are also used in the census (all except Arab). 75% of the census records were for White British, and 95% of these were also recorded as White British in the school records. The remaining 5% were mainly recorded as ‘Other White’ or were missing (2% each). The reliability of each other category was lower than 95%. Asian categories Indian, Bangladeshi, Pakistani and Chinese had between 83% and 92% remaining in the same category in the school records. Black categories African and Caribbean had 83% and 77% respectively. Mixed categories, White with African, Caribbean or Asian, had between 54% and 62%. Other categories had 50% or less remaining in the same category. Overall, 13% of all children had a different category in the school census from the category in the 2011 census. The ONS summarized that

‘a high level of agreement between the ethnicity on the 2011 Census and school census was observed although this did differ by ethnic group’

(Office for National Statistics (2014a), page 37).

Mathur *et al*. (2013) showed that 85% of records on two major English health databases had the same ethnic group recorded, whereas Saunders *et al*. (2013) found only 4.9% discordance overall between self‐reported ethnicity and ethnicity recorded on hospital episode statistics records for English cancer patients. However,

‘for many major ethnic groups (“Indian”, “Pakistani”, “Bangladeshi”, “Chinese”, “Black‐Caribbean” and “Black African”), routine hospital data will miscode between 20% and 35% of all patients who self‐report that they belong to these ethnic groups’

(Saunders *et al*. (2013), page 5).

Kesler and Schwartzman (2014) have provided a recent example of studies that associate ethnic categories with social characteristics. In their evidence children of immigrants with higher socio‐economic status tend to choose more often a White British category of ethnicity than others of the same overseas country of origin. However, the children's own socio‐economic status tended to be associated in the opposite direction; those with higher economic attainment are more likely to claim a minority status. Such choices were more ‘available’ and more often taken by those with European origins. Although not directly addressing the reliability of ethnic categories, Kesler and Schwartzman and their quoted antecedent studies do show the room for choice and changes in ethnic identity.

Very few studies have reported the same people's response to a repeated question on ethnicity. Many countries in the Americas have used race, colour or ethnic origin questions, but an analysis of their reliability is lacking. Correspondence with the census bureaus of the USA, Canada and Australia identified no such studies, but a journal referee pointed out Biddle and Campbell (2015), a product of the recently created Australian census longitudinal data set. They found that about 10% of the Australian indigenous population in 2006 had moved in 2011 to a non‐indigenous category or had not answered the question, whereas those moving in the other direction, into an indigenous category, added about 13% to the indigenous population. A multivariate analysis showed complex associations of movement with family composition and an urban–rural dimension.

In the USA, ancestry was introduced to the census in 1980. The results for White residents showed a simplification over the life course according to Lieberson and Waters (1993). Apart from a tendency for parents to write in only the first of their children's ancestry,

‘all the other puzzling findings we investigated, however, do seem to be reflecting the volatile nature of ethnic ancestry in the U.S.’

(Lieberson and Waters (1993), page 446). These were conclusions from analysing the age and parentage of respondents, rather than a direct comparison of the same question asked on more than one occasion.

In this paper we shall see whether the considerable level of unreliability between different sources for ethnicity is repeated in the census for England and Wales. We examine evidence from the LS for England and Wales for individuals’ census responses in 2001 and 2011, evidence from the 2011 Census Quality Survey, which linked census responses to the same questions in a sample survey, and evidence from the Labour Force Survey whose panel structure allowed ethnic group to be asked more than once of the same sample. We compare our results with those of a similar study for census responses between 1991 and 2001 (Simpson and Akinwale, 2007).

## 3 Sources of data

The core analyses of this paper compare the same individuals’ ethnic group across more than one census, using the ONS LS. This systematic sample from each population census in England and Wales since 1971 takes all respondents with one of four birth dates, linking their records across time together with information from health service registrations and vital statistics systems (Blackwell *et al*., 2003; Office for National Statistics, 2014b). We examine in particular the records linked between the 1991, 2001 and 2011 censuses and focus on the second decade. The linkage of individual records across censuses was pioneered in England and Wales and in France (Institut National de la Statistique et des Etudes Economiques, 2014), though more recently there have been similar studies in Scotland, Northern Ireland, Australia and New Zealand, and similar demographic data sets may be based in other countries on administrative data. The LS contains approximately half a million usual residents in each census. The proportion of the population linked across the censuses depends on births, deaths and migration during each decade, as well as response rates to the censuses and the success of linkage, as quantified for each ethnic group in Section 4.

In each census, questions that are missing or invalid on a returned questionnaire are imputed, and a flag retained on the LS for each of 2001 and 2011. For our analysis in Sections 5 and 6 we include values for ethnic group that have been imputed, better to understand the effect of all sources of unreliability on published census output of ethnic group, and study the effect of imputation more directly later in the paper.

To minimize the risk of disclosure of personal information, all counts of fewer than 10 people from the LS are represented by the symbol ‘—’, and these counts are excluded from the totals of the tables in which they are found. Where possible, rates and proportions have been computed from unpublished tables without suppression of small counts to give greatest accuracy. For this reason, numbers will not match in every table. In all our analyses we include only those who were resident, and we exclude visitors. We have reported neither statistical modelling nor formal tests but have given sample sizes to indicate the robustness of comparisons between proportions on which the analysis is based.

We shorten the terms used on the census form for ethnic group, e.g. using simply ‘Pakistani’ instead of the heading and subheading ‘Asian/Asian British: Pakistani’. We shall often use White British to refer to the ‘White: British’ of the 2001 census, the ‘White: English/Welsh/Scottish/Northern Irish/British’ of the 2011 census in England and the ‘White: Welsh/English/Scottish/Northern Irish/British’ of the 2011 census in Wales. Each usage will be clear from the context and by reference to the ethnic group questions that have been reproduced in Fig. 1.

To help to interpret the results from these analyses of change in ethnic group across a decade, we draw on two other analyses of change in recorded ethnic group undertaken close to the time of the 2011 census. First, the 2011 Census Quality Survey repeated the census questions 3 months after the census enumeration, by face‐to‐face interviewing of a sample of respondents (Office for National Statistics, 2014c). It distinguished proxy responses to the survey from personally completed information and asked whether the census had been completed by the person concerned. Second, the quarterly Labour Force Survey has a rotating panel, each individual remaining in the sample for four quarters. In the first quarter of 2011 it asked all its respondents the 2011 census ethnic group question, allowing comparison with answers to the 2001 census ethnic group question that was asked previously of those who were continuing in the sample from the previous four quarters (Milburn, 2013). This study excluded those whose record was obtained by proxy from another household member. Although both these studies include unreliability induced by a different mode of collection (paper, phone or face to face), their results on short‐term reliability and the effect of proxy reporting are helpful in the interpretation of reliability across a decade. A third study, the 2011 Census Coverage Survey, recorded the ethnic group for a large follow‐up sample but implemented a substantially different approach to the question whose output is clearly biased towards residual categories; for this reason its outputs are not used in this study (Office for National Statistics, 2013a).

## 4 Population turnover for each ethnic group: linkage of census records 2001–2011

**Figure 2:**
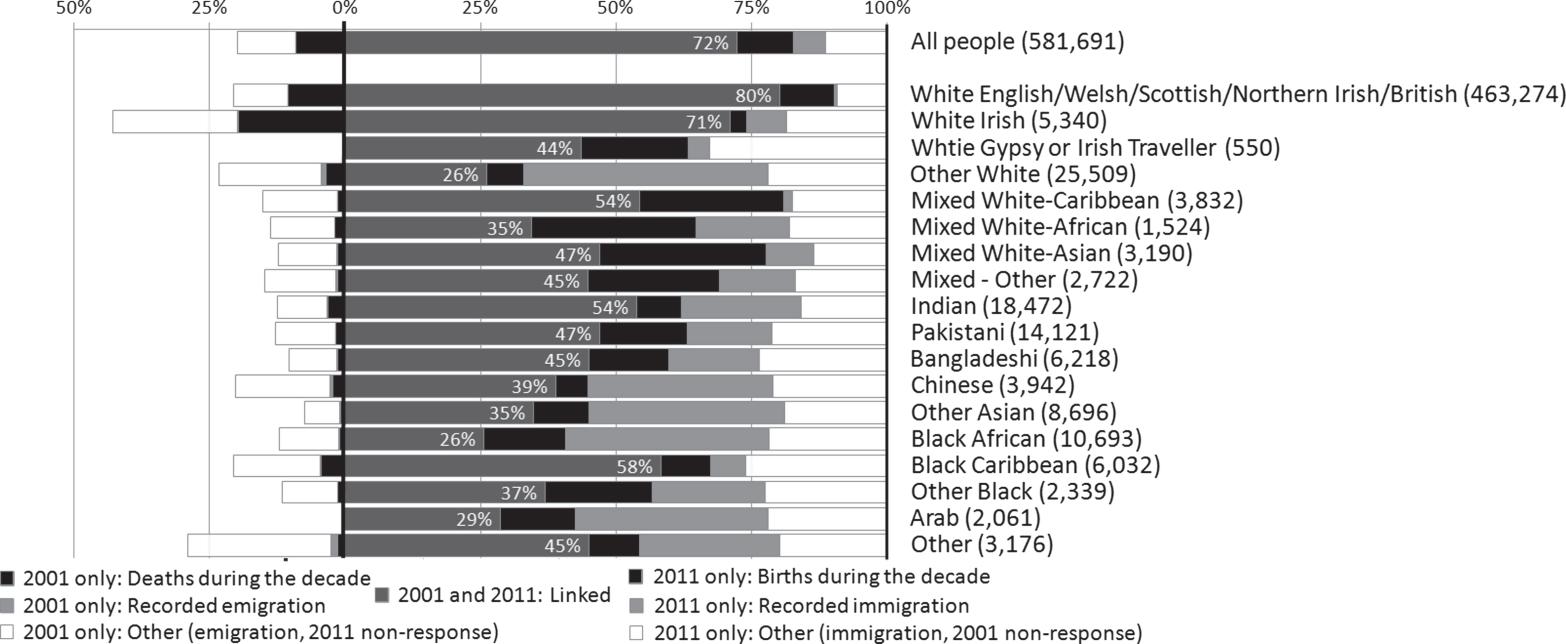
How the population of each ethnic group changed 2001–2011: linkage and reasons for non‐linkage (source, ONS LS, an approximately 1% sample; 2011 sample sizes are in parentheses; all usual residents who were enumerated in either 2001 or 2011; the light grey areas are recorded emigration and immigration; the white areas are unlinked individuals who are assumed to have emigrated, or immigrated or to be non‐respondents; the ethnic group is as in the 2011 census, except for those only in the 2001 population, for whom it is as recorded in the 2001 census)

Fig. 2 summarizes the relationship between the 2001 and 2011 populations, based on linking 2001 and 2011 census records from the 1% sample LS. The population that was enumerated by the census in 2011 is represented by the main block in Fig. 2, between the marks for 0% and 100%. Those who were enumerated in 2001 but not linked to any census record from 2011 appear to the left of the main block.

Most groups other than White British and White Irish can be seen in Fig. 2 to have a relatively high proportion of their 2011 population born during the decade, a lower proportion of deaths during the decade and higher levels of immigration and emigration. Where ‘immigration or non‐response’ is relatively high this represents unknown reasons for someone enumerated in 2011 not being linked to a 2001 record. It is likely to include many who were not enumerated in 2001 but did respond in 2011. The rate of non‐response to the 2001 census varied between ethnic groups more than twofold and was least for White British. Similarly, the higher ‘emigration or non‐response’ represents records from 2001 that were not linked to 2011 with unknown reason and is higher in most ethnic minority groups, which also had higher non‐response in 2011 (Office for National Statistics, 2012a).

Ethnic groups with a relatively high proportion of births, or of immigrants or of non‐response in 2001, have a smaller proportion enumerated in both censuses. All ethnic minority groups have fewer than three‐quarters of their 2011 population also in the 2001 population. For the Arab, Black African and ‘White: Other’ groups, the proportion in both censuses is less than a third. The White Irish group for example has relatively few births but considerably more immigration than the average during the decade, so its proportion of the 2011 census linked to 2001 is 71%, compared with the White British 80%.

Two points of caution should be made when interpreting this evidence. The two new tick boxes added for the first time in the 2011 census, ‘Arab’ and ‘White: Gypsy or Irish Traveller’, do not have an equivalent in 2001. Therefore the LS cannot quantify their deaths, emigration and other reasons for exit during the decade. More pertinently for the remainder of this paper, when a person's recorded ethnic group changes from one census to the next, this will change the bar to which they are allocated in Fig. 2. The deaths and emigration represented on the left of Fig. 2 are not exactly comparable with the categories that they are shown against to their right.

## 5 Reliable comparisons of ethnic group across time

### 5.1 Reliability of individuals’ ethnic group and its effect on group population

For the individuals who were enumerated in both censuses and linked in the LS sample, we now examine the degree of stability for each ethnic group, giving the complete matrix of individuals’ ethnic group in 2001 and 2011, and measures of stability for each ethnic group and for aggregates of groups.

Table 1 is the complete matrix of ethnic group as recorded for individuals who were enumerated in both 2001 and 2011. For example, of the total sample of 4069 people who were in both censuses and identified as ‘White: Irish’ in 2001:

**Table 1:** Ethnic group 2001 and ethnic group 2011†

*Ethnic group 2001*	*Counts for the following ethnic groups in 2011:*																		*Totals for 2001*
	*White*				*Mixed or multiple ethnic groups*				*Asian or Asian British*					*Black, African, Caribbean or*			*Other ethnic group*		
														*Black British*					
	*1, English/*	*2, Irish*	*3, Gypsy*	*4, Other*	*5, White and*	*6, White and*	*7, White and*	*8, Other*	*9, Indian*	*10, Pakistani*	*11, Bangladeshi*	*12, Chinese*	*13, Other*				*17, Arab*	*18, Any other*	
	*Welsh/*		*or Irish*	*White*	*Black*	*Black*	*Asian*	*Mixed*					*Asian*	*14, Black*	*15, Black*	*16, Other*		*ethnic group*	
	*Scottish/*		*Traveller*		*Caribbean*	*African*								*African*	*Caribbean*	*Black/*			
	*Northern*															*African/*			
	*Irish/*															*Caribbean*			
	*British*																		
*White*																			
1, White: British	*366144*	689	216	1539	423	71	252	315	170	144	75	35	89	67	141	48	47	204	370669
2, White: Irish	1008	*3026*	—‡	24	—‡		—‡	11	—‡					—‡	—‡			—‡	4069
3, Other White	2373	38	15	*4785*	17	12	62	166	35	14	—‡		82	36	18	12	127	308	8100
*Mixed*																			
4, White and Black Carribean	210	—‡		14	*1372*	17	—‡	65		—‡		—‡		—‡	72	17		—‡	1767
5, White and Black African	57	—‡		12	22	*332*	—‡	48	—‡					58		—‡	22	10	561
6, White and Asian	218	—‡		38	10	—‡	*877*	94	36	49	16	—‡	73		—‡	—‡	26	36	1473
7, Other Mixed	185	—‡		71	87	30	117	*328*	22	—‡	—‡	17	74	19	13	14	37	79	1093
*Asian or Asian British*																			
8, Indian	77	—‡		17	—‡		37	10	*9033*	69	37	14	540	12	—‡	11	—‡	386	10243
9, Pakistani	79	—‡		17			28	—‡	86	*6040*	34	—‡	243	—‡	—‡	—‡	—‡	11	6538
10, Bangladeshi	30						—‡	—‡	12	20	*2556*	—‡	11					—‡	2629
11, Other Asian	43			35	—‡		35	26	342	193	35	—‡	*1007*	10	12	16	119	143	2016
*Black or Black British*																			
12, Black Caribbean	88	—‡		16	113	—‡	—‡	32	14		—‡		23	35	*2929*	330		13	3593
13, Black African	41	—‡		20	—‡	35	—‡	14	34	—‡	—‡		31	*2383*	32	193	28	31	2842
14, Other Black	40	—‡		—‡	17	—‡		33	—‡	—‡	—‡		—‡	79	258	*193*	11	—‡	631
*Chinese or other*																			
15, Chinese	38	—‡		—‡			14	18	—‡	—‡		*1386*	38		—‡			—‡	1494
16, Any Other	62	—‡		49	—‡	—‡	39	43	25	16	—‡	48	784	—‡		—‡	158	*173*	1397
Totals for 2011	370693	3753	231	6637	2061	497	1461	1203	9809	6545	2753	1500	2995	2699	3475	834	575	1394	419115

†Source, ONS LS, England and Wales, usual residents who were enumerated in both 2001 and 2011. Values for ethnic group imputed into enumerated resident records in either census are included. Cells in italics show those people who were recorded in both censuses with the same (or very similarly worded) ethnic group.

‡ Cells less than 10, according to ONS rules to minimize the risk of disclosure of personal information. They are excluded from the totals in this table.

- 3026 identified again as ‘White: Irish’ in 2011;
- 1008 identified as ‘White: English/Welsh/Scottish/Northern Irish/British’ in 2011;
- Others were recorded in eight of the other categories. 35 were recorded as Other White or Other Mixed whereas fewer than 10 were recorded in each of six other categories in 2011.

Therefore only 74% of the White Irish of 2001 remained with that category. In contrast, 727 respondents had moved to White Irish in the 2011 census from the White British and the ‘White: Other’ categories in 2001 (and from a few other categories with counts that were too small to show). Overall 3753 were recorded in 2011 as White Irish. The net change from one census to the next due to changes in individuals’ recorded ethnic group was a reduction in the Irish population.

We use three summary measures to describe the reliability of each ethnic group:

- stability (from time 1)—of the total in the category in 2001, the percentage remaining in that category in 2011 (values lower than 100% show instability, people moving away from the category);
- stability (to time 2)—of the number in a category in 2011, the percentage already in it in 2001 (values lower than 100% show instability, people moving into the category);
- marginal change—the 2011 population as a percentage higher or lower than the 2001 population for the same category. This is the net result of the two types of stability that are reflected in the first two measures. Values away from 0% show an overall change in the category's population due to individuals moving between categories.

These three measures are given in Table 2 along with the data from which they are calculated, and the same measures of reliability for the period 1991–2001. The measures have been chosen to reflect the two‐directional nature of movement. Neither census is more valid, so the concepts of sensitivity and specificity from test theory are not relevant, despite also being derived from similar 2×2 classifications. There is more similarity with migration theory and the effect of movement between areas on their populations. The permeability of ethnic boundaries has been referred to as the ‘modifiable ethnic unit problem’ by Mateos (2014), page 248.

**Table 2:** Ethnic group categories: stability for individual responses 2001–2011†

*Group*	*Measures of reliability of each ethnic group 2001–2011*							*Measures of reliability 1991–2001*	
	*2001*	*2011*	*Both years*	*Stability from*	*Stability to*	*Marginal*	*Stability from*	*Stability to*	*Marginal*
				*2001: of 2001,*	*2011: of 2011,*	*change*	*1991: of 1991,*	*2011: of 2001,*	*change (%)*
				*% remaining in*	*% already in*	*(2011/2001)(%)*	*% remaining in the*	*% already in the*	
				*the group*	*the group*		*group in 2001*	*group in 1991*	
				*(both/2001)*	*(both/2011)*				
*White*									
White British (English/	370669	370693	366144	98.8	98.8	0.0			
Welsh/Scottish/									
Northern Irish /British)									
Irish	4102	3788	3026	73.8	79.9	−7.7	99.5	99.6	−0.2
Other	8106	6654	4785	59.0	71.9	−17.9			
*Mixed*									
White and Black Caribbean	1795	2076	1372	76.4	66.1	15.7			
White and Black African	584	520	332	56.8	63.8	−11.0			
White and Asian	1491	1486	877	58.8	59.0	−0.3			
Other	1109	1212	328	29.6	27.1	9.3			
*Asian*									
Indian	10262	9832	9033	88.0	91.9	−4.2	91.0	93.7	−2.8
Pakistani	6573	6569	6040	91.9	91.9	−0.1	91.9	92.8	−0.6
Bangladeshi	2649	2781	2556	96.5	91.9	5.0	93.4	94.8	−1.5
other	2025	2999	1007	49.7	33.6	48.1	33.6	35.1	2.7
*Black*									
Caribbean	3611	3503	2929	81.1	83.6	−3.0	77.2	82.8	−6.8
African	2866	2722	2383	83.1	87.5	−5.0	77.4	83.0	−6.7
Other	662	857	193	29.2	22.5	29.5	8.3	20.0	−58.3
*Other*									
Chinese	1524	1522	1386	90.9	91.1	−0.1	91.0	88.6	−4.2
Any Other	1427	1417	173	12.1	12.2	−0.7	7.0	19.1	−63.4
Gypsy or Irish Traveller		240							
Arab		584							
All people	419455	419455	402564	96.0	96.0	0.0	98.0	98.0	0.0

†Source: ONS LS, England and Wales, usual residents who were enumerated in both 2001 and 2011. This table is calculated from a version of Table 1 without suppression of small number cells, to provide more accurate indicators than if calculated directly from Table 1. The counts are therefore slightly higher than in Table 1. The indicators for 1991–2001 are calculated from Simpson and Akinwale (2007), Table 5. Categories are matched with those using the same labels in each census except ‘White: British’ in 2001 is matched with ‘White: English/Welsh/Scottish/Northern Irish/British’ in 2011. Gypsy or Irish Traveller, and Arab are new categories in 2011 and have not been matched with any of those in 2001 for the calculation of stability.

96.0% of the 2001 population that was enumerated again in 2011 reported the same or equivalent ethnic group category. This level of stability was less than the 98.0% for the period 1991–2001 (‘All people’ in Table 2). Put the other way, twice the proportion changed ethnic group categories between 2001 and 2011 than the proportion between 1991 and 2001, in spite of the fewer changes to the question. This is partly due to the growth of minority groups which all had lower stability than the White or White British groups in both periods.

**Figure 3:**
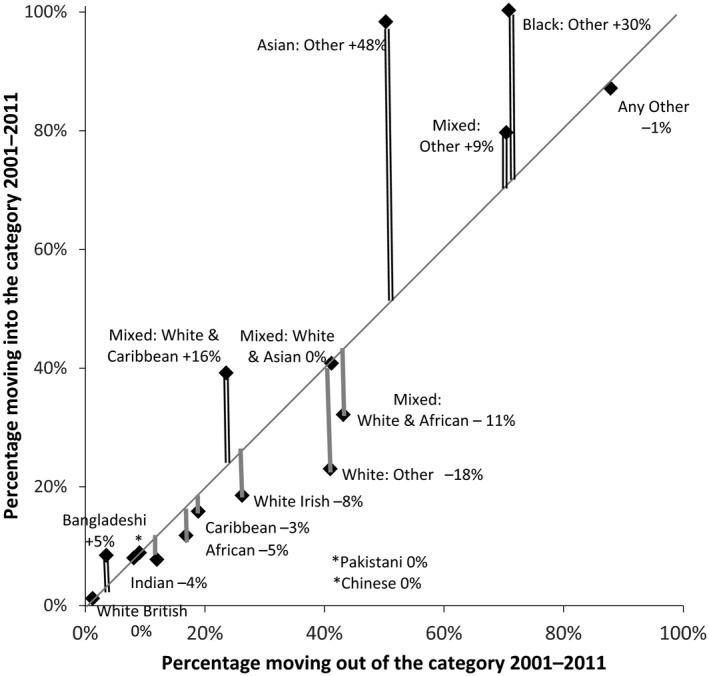
Percentage moving into and out of each category, and net transfer 2001–2011 (source, ONS LS, England and Wales, usual residents who were enumerated in both 2001 and 2011; the vertical lines and the figure next to each label show the net transfer towards the group, the difference between the percentage moving in and moving out of the group; all percentages are of the 2001 population

The level of stability varied greatly between ethnic groups in the period 2001–2011 as it had in 1991–2001 and was again particularly low for residual, ‘Other’, groups. Fig. 3 plots the moves into and out of each category for 2001–2011, so that the distance from the diagonal shows the marginal change, a net gain or loss from the moves.

The White British category shows the highest stability. 98.8% of its 2001 population that were enumerated again in 2011 reported the equivalent category, ‘White English/Welsh/Scottish/Northern Irish/British’. All the Asian categories Indian, Pakistani and Bangladeshi and Chinese are less stable (88–97%) than the White British category but more stable than other categories. The Chinese category moved between broad categories on the census form, from ‘Other’ to ‘Asian or Asian British’ (see Fig. 1), but maintained the same stability that it had had in the 1991–2001 period: 91% of those who had been recorded as Chinese in one census remained as Chinese in the next census.

From Table 1, residents recorded as Indian and Pakistani in 2001 but not in 2011 are most likely to have moved into Other Asian in 2011 (5% and 4% respectively); fewer moved in the opposite direction. In contrast, more people joined the Bangladeshi category than left it, so the group increased its size by 5% from the net movement of individuals who changed their ethnic group.

The Black categories Caribbean and African are less stable (81% and 83% respectively) than the Asian categories but more so than during 1991–2001 (each 77%). This is probably due to the inclusion of Mixed categories in 2001, which caused some of the instability in the Black categories during the earlier decade.

The Mixed categories ‘White&Caribbean’, ‘White&African’ and ‘White&Asian’ are less stable than the ‘single’ categories discussed so far (76%, 57% and 59% respectively). The nature of this instability varies. From Table 1, the ‘White&Asian’ in 2001 were more likely to move in 2011 into White British, than were ‘White&Caribbean’ and ‘White&African’. The ‘White&Caribbean’ in 2001 were less likely to move into another Mixed group than were the other Mixed groups. Moreover, ‘White&Caribbean’ in 2011 were more likely to be added to *from* those who were White British in 2001 rather than to contribute to it. All in all, these movements result in highest stability for the ‘White&Caribbean’ group and an addition of 16% to its numbers by net movement from other groups.

The White Irish group also has a relatively low stability, at 74%. The great majority moved from or to the White British group (Table 1). The White Irish group lost more than it gained: a net loss of 8% simply from the same population switching ethnic groups.

The residual ‘Other’ categories are particularly unstable. ‘White: Other’ is the only residual category where as many as half remained in that category from 2001 to 2011. In contrast ‘Mixed: Other’, ‘Asian: Other’ and ‘Black: Other’ each grew from net changes in individuals’ ethnic group, by 9%, 48% and 30% respectively. This growth is at odds with the period 1991–2001 when the ‘Black Other’ and ‘Other’ categories were reduced because many people in them moved to the new Mixed categories and the new ‘White: Other’ category.

The ‘Asian: Other’ category has been particularly affected by changes of question since the ethnic group question was first introduced. In 1991 it was a category that was entirely constructed from answers written into the final ‘Other’ option on the form, whereas from 2001 it has been a separate category that could be chosen by respondents. In spite of the loss to Mixed groups in 2001, and although only a third of its 1991 population remained with it, the category grew from others who chose it for the first time in 2001 (Simpson and Akinwale, 2007). In 2011, the Chinese category was moved into the Asian broad heading on the census form, and many chose ‘Asian: Other’ who had not done so before, including substantial numbers who were previously in the Indian and Pakistani groups. The ‘Asian: Other’ growth of 48% simply from a net transfer from other categories is nonetheless much smaller than its growth from net immigration and an excess of new births over deaths. Overall its population more than trebled in the period 2001–2011.

The ‘White: Other’ category reduced in size by 18% as a result of moves to other groups, in contrast with the growth of other residual categories. The main exchange was with the White British group. Nearly a third of those who chose ‘White: Other’ in 2001 had moved to White British in 2011. Fewer moved in the opposite direction. The switching towards White British has two clear candidate causes. First, the relabelling of the White British group as ‘White—English/Welsh/Scottish/Northern Irish/British’ in 2011 meant that those who felt strongly about their country identity could more comfortably place themselves in the White British group. Second, the tendency to move towards the perceived identity of the establishment is more easily achieved by White than other groups, as noted for children by Kesler and Schwartzman (2014). But there was also a considerable net loss from ‘White: Other’ to non‐White groups which must have other causes. The overall loss of 18% of the ‘White: Other’ ethnic group from category switching is small in the context of its overall growth of 80% in the census output from 2001 to 2011, in particular due to overseas migration from Eastern Europe during the decade.

If pairs or groups of categories swap people between them when the ethnic group question is repeated, it suggests that the boundary between those categories is not clear for those answering the question. Amalgamating some categories does give more stability to comparisons over time (details are provided in Simpson *et al*. (2014)). A little less than half the movement between individual categories is due to movement within broad categories. The broad categories therefore have greater stability than some or all of their constituent individual categories: White (99.2%), Mixed (68.6%), Asian (94.3%), Black (90.1%) and Other (11.4%). However, the gains in stability are small and must be weighed against the cost of losing the detail of ethnic group that is the purpose of the question. The improvement is not sufficient either to invalidate the separate identity of each category, nor to suggest that a comparison of ethnic groups from the census in 2001 and 2011 should use the amalgamated groups. A similar conclusion was reached for the decade 1991–2001, when in particular the Mixed categories were candidates for including with the ‘single’ categories for comparison across that decade. But no such amalgamations were successful in producing reliable comparisons (Simpson and Akinwale (2007), page 203).

### 5.2 Recommended comparisons of ethnic categories across time

It is considered that the groups with stability across time of less than half, despite the same or similar category labels, should not be compared without the substantial caution that their composition has changed not only due to migration, births and deaths but also because of many moves to and from other ethnic group categories.

For comparisons of ethnic group populations across the three censuses of 1991, 2001 and 2011, the seven categories White, Caribbean, African, Indian, Pakistani, Bangladeshi and Chinese were found to be the most stable when individual responses to the 1991 and 2001 censuses were compared (Simpson and Akinwale, 2007; Office for National Statistics, 2006). This paper finds that they continue to have relatively high stability between 2001 and 2011. ‘White’ is that named category in 1991, and all the categories in the ‘White’ heading in 2001 and 2011. The other six categories are alone in each of 1991, 2001 and 2011. All the remaining ‘Other’ categories and ‘Mixed’ categories have been omitted in this three‐census comparison. They can be presented as an eighth ‘Other’ residual category for completeness, but this residual category changes in its composition and therefore is not comparable over time. Its lower reliability should always be clearly indicated and changes in its socio‐economic attributes have little meaning.

For comparisons of ethnic group populations across the two most recent censuses of 2001 and 2011, the 12 categories with higher stability than 50% are recommended. As there were more categories in both 2001 and 2011 than in 1991, with fewer changes than in the previous decade, one can include more than the seven comparable categories that are available for a 1991–2011 comparison. In particular the Mixed ethnic group categories introduced in 2001 were unchanged in 2011 and can be compared either as a whole or as the individual categories. These 12 most reliable categories, each with stability of over 50% whether calculated as a proportion of the 2001 or 2011 population, are White British, White Irish, ‘White: Other’ (include ‘White: Gypsy or Irish Traveller’ in 2011), Mixed White–Caribbean, Mixed White–African, Mixed White–Asian, Indian, Pakistani, Bangladeshi, Chinese, Caribbean and African.

The other four residual ‘Other’ categories have much lower reliability, with less than half remaining in the same category in the two censuses, and this low reliability should be clearly indicated if they are used to report comparisons between 2001 and 2011. As for analyses from 1991 to 2011, these residual categories may usually be omitted from analyses of change over time, or amalgamated into one category to provide the residual that adds to a population total, with its lower reliability clearly indicated; changes in its socio‐economic attributes or those of its individual component categories have little meaning. The Arab category from 2011 should be included in this residual category when making comparisons across time.

## 6 Sources of unreliability of ethnic group

In this section, we attempt to quantify the sources of unreliability that have been described above. Having acknowledged that the concept of ethnicity lacks objectivity or any essential character that would remain fixed for every individual, it is tempting to interpret individual changes in ethnicity as chosen changes in identity, in response to societal, political, family or other contexts which the individual experiences. Such conscious changes of ethnic group would, we argue, accumulate in number over a longer period of time. Thus they would be more noticeable between two successive censuses than between two responses closely following each other. But here we find survey evidence that reliability over a few months is no more than reliability over the decade between the 2001 and 2011 censuses. From this we conclude that changes in ethnic group are much more likely to have technical and behavioural reasons that do not involve a conscious shift in identity.

Table 3 lists the seven possible reasons for a change in an individual's ethnic group. It provides an estimate of the percentage of people who changed ethnic group for each reason, out of all people who changed their ethnic group between 2001 and 2011. The contribution of some sources of change can be estimated directly, whereas for others less precise estimates are made. The remainder of this section provides a discussion of the evidence for these estimates.

**Table 3:** Estimates of reasons for change in a person's ethnic group, 2001–2011†

*Source of a change in recorded ethnic group*	*% of changes*
	*2001–2011*
1, The census question has changed since it was introduced	10–15
	
2, Imputation: when the question has not been answered, an answer is estimated or imputed by the census offices	19
	
3, Errors in the census processing	2.6
4, Changes in the allocation by census offices of write‐in response	About 0.5
	
5, Proxy reporting: a different person filled in the form at each census	10–15
	
6, A conscious change in identity	5–10
7, More than one response is suitable for	Estimated as
the same person	a residual,
	38–53

†Estimates derived in the text.

The number of categories has grown each time that the ethnic group question has been repeated in the census (Fig. 1). Our first estimate of the effect of a change of question on individuals changing their recorded ethnic group is 4.8%, calculated simply from the LS as the proportion of its members’ 16891 changes of ethnic group between 2001 and 2011 which involved a move to Arab or to ‘Gypsy & Irish Traveller’, the new categories in 2011 that were not in the question in 2001. From 1991 to 2001 the proportion of moves that were to one of the four Mixed groups that were new in 2001 was 39.2% (Simpson and Akinwale (2007), Table 5).

These estimates of the effect of changes of question must be treated as minimums, because they do not include effects of changes to the question's format, to the wording of a category that otherwise did not change or the order of the categories. In 2011, Chinese was included under the broad ‘Asian/Asian British’ heading rather than the final ‘Other’ heading as in 2001. It is likely that this had an influence on non‐Chinese from eastern Asia, to encourage them to record themselves in the write‐in box under ‘Asian/Asian British’. Indeed, more than half of all those who had been recorded as ‘Chinese or Other: Other’ in 2001 had moved to ‘Asian/Asian British: Other’ in 2011 (Table 1: 784 of 1397), or 4.6% of all people who changed categories. Another change in format involves the positioning of ‘British’ and ‘Irish’ labels in one row under the heading ‘White’ in 2001, which raises the possibility that the box between the two labels was ticked by some intending to identify as ‘White: British’ whereas that box would be processed as ‘White: Irish’ (Fig. 1). Indian and Pakistani, and Caribbean and African, were also on a single row in 2001 but not in 2011. Our analysis of the LS shows that a little over half of the sample who moved from White Irish in 2001 to White British in 2011 (1008 in Table 1) were not born in Ireland or Northern Ireland and recorded themselves as only with English, Scottish, Welsh or British national identities. This suggests that the shift away from White Irish that was noted above and based on a sample in 2011 of 314 fewer than in 2001 (Table 2) may have been partially or even wholly due to an overestimate in 2001 caused by the format of the question. Another major change was the wording of the first category under the ‘White’ heading, which changed from ‘British’ to ‘English/Welsh/Scottish/Northern Irish/British’. This change may have led to some of the moves to it from ‘White: Irish’ and ‘White: Other’.

Our estimate for the overall effect of question changes on moves between ethnic groups 2001–2011 is therefore a range, 10–15% of all moves, which is considerably higher than the 4.9% who moved to the two new categories.

The effect of imputation is straightforwardly estimated as 19.1% from the LS: out of all who changed ethnic group, the proportion whose category was imputed in either 2001 or 2011. This is a slight overestimate, as some of those who were imputed would have changed group even if they had been recorded directly without imputation.

Errors in the automated data processing were estimated by the ONS for ethnic group as 1.2% in 2011 and 1.4% in 2001, from a sample which was manually checked (Office for National Statistics (2012b), page 9). Error at either census would create a change in ethnic group between the two censuses, so our estimate of this source of moves between ethnic groups adds the two estimates. It is likely that these errors are independent, allowing us to deduct the product of the error rates, but this makes no difference to the results at our level of approximation.

Changed allocation of write‐in responses creates some changes of ethnic group. About 25000 write‐in answers of Kashmiri were received at each census. They were allocated to Pakistani in 2001, but to the residual ‘Other’ categories in 2011 (the figures are given in census tables M221bNat of 2001 and CT0100 of 2011). On the reasonable assumptions that the LS sample contains the same proportion of Kashmiri write‐in answers as the population, and that about half were in both censuses (taken from Fig. 2), writing in Kashmiri in both censuses, then this reallocation would account for 80, or 0.5%, of the individuals who changed ethnic group. There were other reallocations but none of such significance, and not all these conditions will hold true, so the total effect of changed allocation of write‐in responses is estimated as 0.5% in total.

The Census Quality Survey reports that, among those adults who gave their own survey response, 5.3% gave a different answer from their census response. 8% of these had a census response given by a proxy (Office for National Statistics (2014d), pages 55–56), which is rather higher than the 5% of all adults who were recorded in the census by a proxy (Office for National Statistics, 2014e). 8% is an underestimate of the total percentage of moves between the two censuses that were due to a proxy response, because it excludes children who are more likely to have been proxy reported and excludes proxy reporting in the second response to the question. Our estimate is therefore that 10–15% of moves in ethnic group were due to proxy reporting.

The sixth reason for moving between ethnic groups is a conscious change of the identity that we record on the census. Our reaction to official enquiries such as the census, and our sense of ethnic identity, may both change over time. For either reason or a combination of the two reasons we may decide to declare our ethnic group differently in the census even when not affected by question changes or the errors discussed so far. If we define conscious changes in identity as having a degree of durability then we would expect to see more such changes of identity when measured over a longer period than the effects of question changes, data processing errors, imputation and proxy reporting that would all be seen equally in the short term and long term. Two official surveys repeated the census ethnic group question to the same people after less than a year, which allows a comparison with the 10‐year stability measured from the LS.

The 2011 Census Quality Survey has already been mentioned, finding 5.3% discrepancies, equivalent to a stability of 94.7% overall. The same ethnic group question was used, so there were no changes of question. Discrepancies were also minimized by excluding missing values (so that no imputation was involved), by collecting data with computer‐assisted interviewing, which avoids errors in subsequent data capture, by excluding survey proxy reporting from the analysis and by gaining responses only 2–5 months after the 2011 Census (Office for National Statistics (2014d), page 102). When excluding census proxies, and including children, the rate of stability rises to 95.7% (Office for National Statistics, 2014f). It is noticeable then that, even when excluding the major measurable reasons for individuals having recorded a change in ethnic group, the level of reliability was not higher but slightly lower in this short‐term repeat of the census than the 96.0% that were found for the 10‐year gap between the 2001 and 2011 censuses.

The Census Quality Survey used face‐to‐face interviews. This change in mode of data collection will have introduced some discrepancies. Although the ethnic group question was aided with a show card using the same wording as in the 2011 census, the interaction with an interviewer will have introduced its own biases that induce a different response from the census. In particular the survey found that the net effect of changes between the census and the survey was to double the number of mixed ethnicity responses and to increase by a half the broad Other category, and to reduce the Asian and Black responses. Although the sample was small, with 9000 individuals matched in the report's analyses, it seems that, despite the attempt to replicate the census questions and prompts exactly, the interviewers in the Census Quality Survey elicited significantly more nuanced responses than did the census.

The Labour Force Survey also asked the census question twice of the same people. The interviewees who were first questioned in each of the four quarterly panels of 2010 were asked the 2001 census question by face‐to‐face interview. All were then asked the 2011 census question by telephone in the first quarter of 2011. Proxy responses were eliminated as far as possible before analysis, as were responses from those aged under 16 years. Milburn (2013) has provided information that allows calculation of a measure of stability as has been used in this paper, for nine categories, with White, Mixed and Black each aggregated to single broad categories. The proportion of the 2001 census categories that did not change when asked the 2011 census categories a year or less later is shown in Table 4.

**Table 4:** Stability of ethnic group from the census during 10 years and from the Labour Force Survey during less than 1 year†

*Group*	*Census, during*	*Labour Force*
	*10 years (%)*	*Survey during less*
		*than 1 year (%)*
White	99.2	99.3
Mixed or multiple ethnic groups	68.6	57.2
Indian	88.0	90.5
Pakistani	91.9	96.2
Bangladeshi	96.5	92.1
Chinese	90.9	85.1
Any other Asian background	49.7	59.9
Black, African, Caribbean, Black British	90.1	90.4
Other	12.1	35.3
All nine categories	97.8	97.5

†The proportion of the group at an earlier time which remains in the same group at the later time. Sources, census 2001–2011 from Tables 2 and 3 above. Labour Force Survey 2010–2011 from Milburn (2013), Table 3a, page 10.

Overall, and with few exceptions in the nine individual comparisons, the stability of ethnic group, when repeated in the Labour Force Survey within a year, is not higher than the stability of ethnic group between two censuses 10 years apart. Again, there was a change of mode between the two responses in the Labour Force Survey, from face‐to‐face interview to telephone interview.

One can conclude that the effect of a difference in mode of collection in both the Census Quality Survey and the Labour Force Survey must be as great as the effect of durable shifts in individual ethnic group identity. This is the reason for suggesting that the effect of such durable shifts is small: not more than 10%. It is estimated in Table 3 as the reason for 5–10% of all moves between ethnic groups between 2001 and 2011.

The final reason for moves is one which we think is most common: that more than one response is appropriate. When someone thinks about their ethnic group and considers that it does not clearly fit just one of the categories offered by the census question, they have a choice. A Turkish origin resident with British nationality may consider themselves ‘White: British’, ‘White: Other’ or ‘Other: Other’. Someone with family origins in Britain and India may consider themselves ‘Mixed: White and Asian’, ‘Mixed: Other’, ‘Asian: Other’, ‘Indian’ or ‘Other: Other’. In both these cases, they may tick a different ethnic group on different occasions without changing their own origins or identity. Their view of their identity may be clear, thought through and unambiguous but not identical with a single census category. The point here is simply that their recorded ethnic group will reasonably change without it being a conscious or durable change of identity.

We cannot directly measure the effect of such identities that the ethnic group categories do not allow for. We estimate it as the remaining instability after other reasons for changing ethnic group have been considered as above, taking into account some of the uncertainty in their estimates. From Table 3, these other reasons account for 47–62% of the moves observed between ethnic groups from 2001 to 2011. This leaves 38–53% due to the ambiguity of identity that the question does not allow for. To the extent that our other estimates may be inaccurate, then this estimate also will be in error. But it is reasonable to conclude that about a third to a half of all instability is due to people for whom more than one response is suitable, and that this amounts to about 2% of the whole population that was recorded in both the 2001 and the 2011 censuses.

## 7 Imputation of ethnic group

We turn now to the effect for analysts of the ONS practice of imputing ethnic group when it is missing or, much less frequently, when it is completed in an invalid way such as ticks in several different sections. In the 2011 census database of residents for England and Wales, ethnic group is imputed on 1.6 million (3.1%) records (Office for National Statistics, 2013b). Table 5 includes only those who responded in 2001, of whom 2.1% were imputed in 2011, the lower figure showing some dependence in response patterns over time. The first columns of Table 5 show that the percentage imputed in 2011 was higher for those who had responded in 2001 as one of the Mixed or residual ethnic groups. These are also the groups that we have found to have had greater instability.

**Table 5:** Stability of ethnic group for records which have been imputed in 2011 but not in 2001: percentage of the ethnic group in 2001 who remained in the same group in 2011†

*Group*	*All not*	*Of these,*	*% (of 2nd*	*Imputed same*	*% (of 3rd*
	*imputed in 2001*	*imputed 2011*	*column)*	*as 2001*	*column)*
White British	363259	7393	2.0	7150	96.7
Irish	4014	82	2.0	35	42.7
Other White	7818	236	3.0	82	34.7
Mixed	4810	183	3.8	15	8.2
Indian	10008	253	2.5	137	54.2
Pakistani	6307	144	2.3	85	59.0
Bangladeshi	2550	42	1.6	16	38.1
Other Asian	1956	95	4.9	22	23.2
Black Caribbean	3465	129	3.7	60	46.5
Black African	2737	82	3.0	41	50.0
Other Black	642	45	7.0	—‡	
Chinese	1493	19	1.3	—‡	
Other ethnic group	1387	70	5.0	—‡	
Total	410446	8773	2.1	7652	87.2

†Source, ONS LS, England and Wales. ‡Count less than 10.

Imputation of values for ethnic group was undertaken with the system CANCEIS, which was developed for the Canadian census, as part of the census processing in the UK before construction of tabular and other output. The method is described in detail in Office for National Statistics (2012c). In brief, for a household with missing ethnic group or other ‘cultural’ variables for any of its members, CANCEIS seeks a ‘donor’ household of the same size with a complete record for these and other key variables such as age. It first assembles a pool of potential donor records that are closest to the household with missing cultural values, ‘closeness’ being measured both geographically and in terms of similar values for other variables. From this pool, a final donor is then chosen with probability proportional to its similarity to the household with missing values, which are filled with the donor values. The method ensures that correlations between variables are used in the choice of the donor, e.g. between ethnic group and country of birth.

The aim of imputation is to provide a complete database of plausible values whose distribution between categories is not biased. But how plausible is the allocation of ethnic group to individual records? Is it sufficiently plausible to include these records in analyses of the census microdata that are available to researchers through the samples of anonymized records (the UK public use microdata samples) and the LS?

We cannot answer this question exactly from the data that we have, as we do not know what individuals would have responded to the ethnic group question if they had done so. However, we can investigate whether the imputed value is the same as that provided in 2001, as in Table 5. The samples are reduced as only a small minority of records were imputed in 2011. The Mixed category has therefore been amalgamated as one group. Most ethnic groups—as defined by those answering the question in 2001, have a proportion with ethnic group missing in 2011 that is higher than the 2.0% for White British. What proportion of these have the same ethnic group as provided in 2001?

The overall message of Table 5 is that imputation is much better than a random allocation of ethnic group. Each group is imputed considerably more often than its proportion in the population (which can be seen from the first columns).

With the best imputation we might hope for a proportion remaining in the same group that is equal to the group's stability shown in Table 2. For White British the stability is 98.8% and the stability of the imputed records from Table 5 is 96.7%. For other groups the stability of imputed ethnic group from 2001 to 2011 is rather less than for its general population; for example the largest minority, Indian, has a stability of 54% for imputed values but 88% for all residents present at both censuses. The relative drop in stability when comparing imputed values with the general population is particularly strong for the smaller groups and those most geographically widely spread—the Mixed groups, Bangladeshi and the residual groups. For these groups there would be fewer potential donors with the same ethnic group.

It is likely that those not responding are, at least on average, those whose ethnic group is most likely to be unstable. Discounting the theoretical possibility that imputation has found in every case the ethnic group that would have been answered, the results nonetheless show the general success of imputation, as well as its frequent failure at the individual level by giving a mismatch with the ethnic group that was provided in the previous census.

How should analysts deal with this evidence of the unreliability of the imputed values of ethnic group in microdata? Flags mark these records so that they may be included or excluded in analyses. The percentage of imputed records for any of the groups is not large, and other analysts have found that results from statistical modelling are only marginally affected by the exclusion of records with imputed ethnic group. However, given the evidence that imputed records add unreliability to analyses, it would be good practice to repeat analyses with and without records with imputed ethnic group, and normally to report analyses without those records.

## 8 Characteristics associated with unstable ethnic group

To understand further the nature of ethnic group stability and how it may change in the future, this final analytical section examines its relationship with other variables. We show that a person's country of birth in the UK is associated with lower stability of their recorded ethnic group, that age has a weak and complex relationship to stability of ethnic group and that other variables have relatively very marginal relevance.

In this section we remove the 4.4% of records which although matched across the 2001 and 2011 censuses had values of ethnic group imputed in one or both censuses, because we are interested specifically in what may be associated with a change in ethnic group. As above, these imputed values account for 19% of the instability.

Table 6 shows the proportion of people staying within the same ethnic group from the 2001 to 2011 censuses, separately for those born in the UK, for those born outside the UK in a country associated with the ethnic group declared in 2001 and for other countries. A country that is associated with ethnic group means African countries for the Black African ethnic group, India for the Indian group, and so on.

**Table 6:** Stability of ethnic group from 2001 to 2011, according to country of birth: percentage of ethnic group in 2001 who remained in the same group in 2011†

*Ethnic group 2001*	*All birthplaces*	*Born in*	*Foreign born,*	*Foreign born,*
	*(%)*	*the UK (%)*	*country associated*	*country not*
			*with the ethnic*	*associated with the*
			*group label (%)*	*ethnic group label (%)*
White British	99	99	Not applicable	87
Bangladeshi	99	97	100	92
Pakistani	94	93	96	84
Chinese	94	90	99	89
Indian	90	89	90	91
Black African	85	82	88	75
Black Caribbean	84	80	92	72
White Irish	75	46	90	76

†Source, ONS LS, England and Wales. Excludes records with ethnic group imputed when missing, in either census. Countries associated with Chinese are China and Hong Kong. Countries associated with White Irish are Ireland only. The sample size for each figure is greater than 350 except for the final column for Bangladeshi (sample 36), African (84), Caribbean (155) and Irish (51).

For each minority ethnic group, those born in the UK are less likely to maintain their ethnic group than those born elsewhere. For example 3% of the Bangladeshis in 2001 who were born in the UK changed their ethnic group in 2011, compared with 1% overall. White Irish were particularly likely to shift to another group in 2011 if they had been born in the UK. As we might expect, among those who were born outside the UK, those born in a country that is associated with the ethnic group label given in 2001 were most likely to stick with it. It is easier to accept and stick with ‘Pakistani’, for example, if one was born in Pakistan.

The exception in Table 6 is the Indian group which has stability that is as high for those born overseas in a country other than India as for those born in India. The census itself shows that the majority of these were born in East Africa, who were 15% of all Indians in 2001 in England and Wales. Most emigrated during a short period of political–ethnic turmoil in the 1970s, particularly from Kenya, Tanzania and Uganda. Their Indian identity is as stable as those who were born in India itself, and indeed slightly more so.

**Figure 4:**
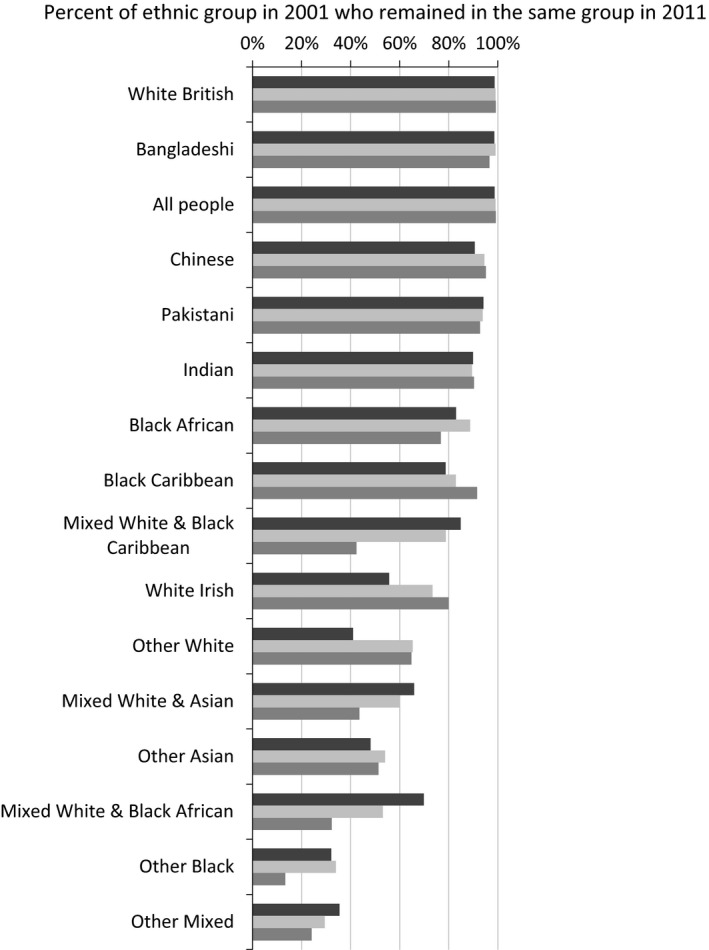
Stability of ethnic group from 2001 to 2011, according to age in 2001 (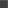, 0–19 years; 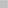, 20–49 years; 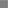, 50 years or older): percentage of the ethnic group in 2001 who remained in the same group in 2011 (source, ONS LS, England and Wales; the ethnic groups are sorted in decreasing order of stability for all people in the group)

How strongly is age related to stability of ethnic group? We would expect younger people to have lower stability, due to the lower stability for UK born among minority groups, and of those whose forms are most likely to be filled by proxy (see above), as would be the case for children in 2001. Fig. 4 shows that the evidence does not uniformly confirm this expectation of less stability for younger ages.

For all four mixed ethnic groups, those aged under 20 years in 2001 were notably more stable in their ethnic group than those at older ages. This was so for younger and older children. It was not a matter only of those leaving the group, as the percentage of those moving into Mixed groups from other groups also increased with age (which is not shown in Fig. 4). It may be that younger people of Mixed ethnicity are more comfortable with the label. This is supported by the research in preparation for the 1991 census that suggested that Mixed ethnicity people preferred to identify with one of their parents’ ethnicities and therefore were not given a mixed option on the census form, whereas by 2001 the Mixed category was deemed more acceptable.

We hypothesized associations of ethnic group stability with other variables, but most showed complex relationships. For the foreign born, longer residence in the UK was associated with more stability as we might expect. People with professional and higher managerial socio‐economic group had, dependent on the ethnic group, either more or less stability, but the differences were not large. For those with White British or Mixed ethnic group in 2001, a language other than English was associated with reduced stability. Also as might be expected, those in a household of people with different ethnic groups in 2001 were more likely to change their group in 2011.

## 9 Discussion

Measures of reliability show how well the ethnic group question categories capture the individual's sense of ethnic identity in a stable way, whether this is an emotionally felt cultural identity, a sense of family origin or simply an acceptance of an official labelling.

Changes of recorded identity occur for various reasons, which between the 2001 and 2011 censuses affected 4.0% of the population who were enumerated on both occasions. A genuine change in an individual's perception of their ethnic identity may occur. However, we have estimated other larger causes of instability in an individual's ethnic group.

Imputation of ethnic group where it is not provided on the census questionnaire is never precise, and these records account for 19% of the changes in ethnic group between 2001 and 2011. Changes of question between the two censuses are associated with 10–15% of changes of ethnic group. Proxy reporting of ethnic group, when a person's details have been provided by someone else, is estimated to lead to another 10–15% of changes of ethnic group. Most other changes are thought to be either because more than one response is a suitable answer for the individual's sense of ethnic identity or due to a weak sense of ethnic identity on the part of the individual. These two subtly different reasons for moving between ethnic groups are estimated to account for between a third and a half of all changes in ethnic group between 2001 and 2011 in England and Wales, because similar levels of change are found when the same census or census‐like question is asked twice within a relatively short time period, as when asked with a decade interval. The finding that the proportion with ethnic group missing is high for those who previously stated a mixed or residual group is another hint that some people find it difficult to place themselves within the categories provided.

Errors in completing the census form that result in an unintended code, errors when scanning hand‐completed forms and changes from one census to the next in the allocation of write‐in responses have each added a small amount of noise to the data that induces some instability.

One might hope that the question will have ‘settled in’ over three censuses, but its overall instability has doubled from 2.0% in 1991–2001 to 4.0% in 2001–2011, in spite of fewer changes to the categories in the second decade. The greater number of people in minority groups, which are least likely to keep to the same category from one census to another, accounts for some of the increased instability, which can therefore be expected to increase further as Britain's diversity continues to grow (Wohland *et al*., 2010).

The number of people without an official ethnic category that directly applies to them is also likely to grow, because of continued immigration from new origins. That three of the ‘Other’ groups (those within the Mixed, Asian and Black broad categories) grew by net transfers from other groups also suggests a growing difficulty with the question.

It could be argued that the question is very good at allowing people to identify in the way that they choose, precisely because the residual ‘Other’ categories are provided and used. But from the perspective of social policy a shift towards the residual categories provides less information and a less reliable indication of trends over time. The question has become less discerning at the same time as there is greater diversity in the population.

The Mixed groups have a strong interchange between censuses with the White groups: both White British and White Other. Although some young people of mixed heritage undoubtedly report as White British, Table 1 above shows that the net change is clearly in the direction from White British to Mixed. Perhaps ethnicity other than White has become more accepted and less stigmatized in the UK, and for that reason more people are unwilling to accept the single categories and choose a mixed category or write in a refined description in one of the ‘Other’ categories.

The longitudinal data that we have used would yield further insights into not only the Mixed and Other categories but also the different factors which shape and change each official ethnic identity, far beyond the capability of the current analyses. What is associated with the net gain to the Bangladeshi group from moves between other groups, and what shapes the net loss to the Indian, African and Bangladeshi groups? The characteristics that influence the choice of one group over another may include not only age and socio‐economic status, but also marriage partner, household structure and the area one lives in and its characteristics. Multivariate modelling methods including panel data regression may better be able to identify factors that are associated with ethnic identity and its change over time, and in that way help to structure our understanding of ethnic fluidity.

Categories in a census may always lag behind the changing diversity of the population and may increase in number in the future to capture the growing diversity, but there is a limit to such a response. At some point in the future, official statistics may be forced to reconsider further the measurement of ethnic group. Other countries’ experiences provide alternatives to help such a reconsideration (Morning, 2008), that include separating colour from ancestry, and allowing free‐text answers (Aspinall, 2012).

In the UK there are also pressures towards increasing use of administrative records in official statistics. There is a relatively large mismatch between ethnicity recorded on census and administrative records, as noted in our review of previous work. This may encourage simplification of recording ethnic group or focus attention on aspects of ethnicity such as religious background, language or countries of family origin, that may be more reliably captured than ethnic group itself.

In the meantime, analysts of the census can pay attention to outputs that give a detailed view of ethnicity, including an amplified list of write‐in answers, and cross‐tabulations of ethnic group with religion, language and country of birth. The ability to commission further cross‐tabulations, and to analyse anonymized microdata from the census, allows a range of research questions to explore ethnicity in more diverse manifestations than the standard 18 categories that were used in 2011.
